# Effects of Virtual Care on Patient and Provider Experience of the Clinical Encounter: Qualitative Hermeneutic Study

**DOI:** 10.2196/52552

**Published:** 2024-11-26

**Authors:** Graham McCaffrey, Erin Wilson, Lela V Zimmer, Anurag Singh, Steinunn Jonatansdottir, Peter Zimmer, David Snadden, Ian D Graham, Martha MacLeod

**Affiliations:** 1 Faculty of Nursing University of Calgary Calgary, AB Canada; 2 School of Nursing University of Northern British Columbia Prince George, BC Canada; 3 Faculty of Medicine University of British Columbia Vancouver, BC Canada; 4 Northern Medical Program University of Northern British Columbia Prince George, BC Canada; 5 School of Epidemiology and Public Health University of Ottawa Ottawa, ON Canada

**Keywords:** virtual medicine, telehealth, professional-patient relations, hermeneutics, kidney, health care facility, British Columbia, Canada, qualitative research, eHealth, health informatics, physician, COVID-19, pandemic, patient experience

## Abstract

**Background:**

Virtual health care has transformed health care delivery, with its use dramatically increasing since the COVID-19 pandemic. While it has been quickly adopted for its convenience and efficiency, there has been a relative lack of in-depth exploration of its human impact, specifically how both patients and providers experience clinical encounters.

**Objective:**

This analysis aims to identify and explore themes of change in how patients and providers in a geographically dispersed renal service described their experiences with virtual care, including those changes that occurred during the COVID-19 pandemic.

**Methods:**

Hermeneutics is an interpretive research methodology that treats human experience as inherently interpretive, generating meaning through interactions with others in specific, historically conditioned, social contexts. A total of 17 patients and 10 providers from various disciplines were interviewed by phone as part of a study on health care implementation in the context of a kidney care service in northern British Columbia, Canada. The interview data were analyzed using a hermeneutic approach, which emphasizes careful attention to reported experiences in relation to the relationships and contexts of care.

**Results:**

During analysis, the interdisciplinary team identified themes related to changes in the clinical encounter and how virtual care influenced perceptions of care among both providers and patients. We organized these themes into 2 categories: the structure and content of the encounter. The structure category included the convenience for patients, who no longer had to travel long distances for appointments, as well as changes in care networks. For example, communication between specialist services and local primary care providers became more crucial for ensuring continuity of care. The content category included issues related to trust-building and assessment. Providers expressed concerns about the difficulty in assessing and understanding their patients’ physical and social well-being beyond laboratory results.

**Conclusions:**

Patients in the study appreciated the convenience of not needing to travel for appointments, while still having the option for in-person contact with local providers or specialists if their condition changed. Providers were more concerned about the loss of visual cues and sensory data for assessments, as well as the reduced opportunity to build relationships through conversation with patients. Providers also described changes in the locus of control and boundaries, as patients could join phone encounters from anywhere, bypassing traditional privacy and confidentiality boundaries. The study offers a nuanced view of the effects of virtual care on clinical encounters in one setting, seen through the experiences of both patients and providers.

## Introduction

Virtual patient-provider encounters have increased dramatically in response to the exigencies of the COVID-19 pandemic. It is not known how virtual care will develop in the future, but it is clear that it has become familiar, even routine, to many providers and patients to a far greater degree than anyone might have expected at the end of 2019. We report on findings from a qualitative study of a virtual kidney care service in northern British Columbia, Canada. The focus of our study was to understand human processes in implementing health care innovation, in this case, the introduction of virtual care by a specialized clinic serving a large geographic area. Our study coincided with the COVID-19 pandemic which meant that work processes and use of technology for virtual care changed again in response. In addition to data about implementation processes, the interviews provided a great deal of information about experiences with virtual care from both provider and patient points of view. We brought these data together under the organizing theme of the clinical encounter, seeing it as a human interaction that is connected to institutional structures, clinical goals, professional roles, and of course, technological means of communication.

Recent literature has begun to explore the implications for health care practitioners and service users of virtual care. Virtual care has been defined as “any interaction between patients and/or members of their circle of care, occurring remotely, using any forms of communication or information technologies, with the aim of facilitating or maximizing the quality and effectiveness of patient care” [1]. A scoping review of 8 studies on remote renal care found overall satisfaction, with convenience, especially reduced travel time for appointments, identified as the primary advantage to patients [2]. Barriers were identified that affect communication during consultations, often making it more functional and hindering discussions of sensitive issues. One related concern was patients’ difficulty in finding a quiet, private space conducive to a confidential medical conversation [2]. Another scoping review of virtual renal care highlighted a shift from older audio-visual technology, which often required clinic visits, to the use of portable personal devices [3]. However, this shift was accompanied by concerns about disparities arising from social determinants of health, as well as access to technology and reliable internet connections—issues that are increasingly the responsibility of individuals rather than provider agencies. Positive outcomes included high levels of patient satisfaction and fewer emergency room visits or hospital admissions [3]. Several studies recommended hybrid approaches, where, over the course of a clinical relationship, there would be a combination of in-person and virtual contacts between providers and patients [2,4,5]. A pair of complementary guides issued by the Canadian Medical Association [6,7] made recommendations for conduct during virtual consultations. One suggestion for physicians was to wear a white coat as a visual reminder of the nature of the meeting, in the absence of office or clinic space [6]. For patients, the recommendation was to find a space where they could be alone and uninterrupted [7]. Overall, experience with virtual care continues to evolve, with changes in communication technology and new clinical applications. The resulting need to adapt to new modes of practice is reflected in the development of toolkits for clinicians and patients [8,9], designed to address issues of privacy, informed consent, equity, and appropriateness in virtual care encounters.

Not surprisingly, there has been a surge of literature addressing the rapid, and mostly unanticipated, adoption of virtual care in response to COVID-19 restrictions. Studies seem to reinforce existing trends, with perhaps greater emphasis on concerns about the challenges of ensuring clear communication during virtual appointments, especially over the phone [10-13]. Bajgain et al [10] reported in a systematic review of 102 studies from 20 countries that measures of patient experience showed greater convenience as a strongly positive factor, although communication was often perceived as more rushed or uncertain. Other studies focused on the implementation process itself [11] or on simple measures of patient satisfaction [12], but the effects on clinical relationships were less clear [13]. Our study, drawing on interview data from both providers and patients and exploring in detail their experiences with virtual care, adds to this emerging literature. Our objective was to understand virtual clinical encounters as a form of relational event, shaped by complex interactions with structures of care and the frames of meaning that participants bring to these encounters.

## Methods

### Study Design

The study used a qualitative, hermeneutic methodology to explore what it means for patients and providers to implement a virtual care service [14]. The subsequent analysis of interviews with patients and providers aimed to understand their experiences with virtual clinical encounters. The study was conducted in northern British Columbia, Canada, in partnership with knowledge users in Northern Health.

The virtual kidney care (Tele-Kidney Care) program [15] is provided through Northern Health, the health authority that delivers health care across the region to 32 communities and 55 First Nations communities. The sparsely populated region covers an area of 617,271 square kilometers—approximately the size of France—with a population of about 300,000 [16]. The kidney care clinic is located in the regional center, a city with a population of 80,000. Virtual care is offered to individuals with predialysis chronic kidney disease and those who have received kidney transplants. Patients attend their local health care facility for virtual appointments and communicate via video with the specialist kidney care staff located in the regional center. Some follow-up communication, particularly during the COVID-19 pandemic, occurred via telephone.

Study participants were 10 providers and 17 patients, all recruited through the kidney care program. Providers (2 men and 8 women) consisted of 8 clinical professionals (a nephrologist, nurses, a dietitian, a pharmacist, and a unit assistant) and 2 information technology specialists. The patients, 6 men and 11 women, were identified by the program’s clinical leader as individuals who had provided feedback on virtual services to the kidney care team. They were contacted by the clinical leader in person or by telephone and provided with both oral and written information about the study. Those who chose to participate then contacted the research coordinator directly or consented for a care provider to pass their contact information to the coordinator, who then reached out to them. Potential participants were given further information, and if they chose to proceed, written consent was obtained. All providers and patients who were approached contacted the researchers and volunteered to be interviewed. None withdrew from the study.

Two senior authors (GM and MM) and a PhD research trainee (SJ) conducted 30- to 60-minute interviews by telephone between November 2020 and February 2021. Questions for providers focused on delivering kidney care virtually and how this changed with COVID-19. Questions for patients focused on their initial experience with virtual care for kidney treatment and how they had experienced virtual care since then. The interview questions for patients and providers are presented as Multimedia Appendices 1 and 2, respectively. All interviews were audio recorded and transcribed.

Hermeneutics is a qualitative research methodology rooted in the philosophical tradition of interpretation. It views human experience as emerging from the interrelations between individuals and their social environments, which are shaped historically over time [17]. Understanding of experience is developed through dialogue, where differing perspectives lead to new insights into a topic of interest. These principles have been operationalized in research using interviews [17] to address complex topics of human interrelationships, which are influenced and shaped by contextual factors. In the case of the renal service, geography and technology had significant impacts on how care was structured, delivered, and perceived. Data analysis in hermeneutic research goes beyond identifying themes from interview transcripts to develop interpretations that deepen the understanding of the topic in relation to transdisciplinary sources, revealing aspects that may be significant for practice. Hermeneutics is a suitable methodology for exploring a topic like virtual care encounters, which are relatively new and complex, and require investigation into their emerging implications for practice.

The research team analyzed the data through a systematic process, working iteratively through several stages of interpretation. These interpretations were compared, contrasted, and refined with insights from relevant literature and materials. Initial analysis was conducted by 6 academically based team members, with interpretations carried out by the full team. The team discussed the interpretations while remaining open to questions and alternative viewpoints. They included substantive quotes to preserve the depth and context of the data, ensuring rigor and allowing readers to assess the relevance of the findings in the context of virtual care encounters beyond the specialty focus of renal care.

### Ethical Considerations

Harmonized ethical approval for this study was obtained from the University of Northern British Columbia and Northern Health (REB H20-02263). Informed consent was received from all participants, allowing for interpretive analysis of results beyond the original scope of the implementation study. All interview data were deidentified, with identifying details omitted from the reported data. Quotations were organized by theme, not by association with a particular participant. Each participant received a CAD $25 (US $17.86) gift certificate for a coffee shop chain.

## Results

### Exploring Patient and Provider Perspectives on In-Person and Virtual Care Encounters

When the research team met to analyze the data, we found that participants had a lot to say about their experiences with in-person encounters compared with virtual encounters, as well as video-link versus telephone encounters. What made the data so rich was that we interviewed patients who had experienced both in-person and virtual appointments at different stages of their kidney illness and treatment, and who saw these encounters change again during the COVID-19 pandemic. Combined with interviews with providers, we gained a comprehensive and varied perspective on clinical encounters related to chronic kidney disease. Through team discussions, we identified 2 broad themes.

The first theme focused on the structure of the encounter in a phenomenological sense—how 2 or more people connected around a specific occasion of health care needs, and the networks of human, technological, and material resources, required by both patients and providers, that were invoked in order for encounters to happen. The second theme focused on the content of the encounters—how relationships were formed, cultivated, or inhibited through different modes of interaction between patients and providers. Crucially, in the health care context, it also examined the role of trust in clinical encounters.

### Structure of the Encounter: Health Care Systems, Peer Support, and Virtual Network

The rationale for adopting virtual care in regions like northern British Columbia is driven by factors such as distance, low population density, and limited availability of specialized health care resources. Patients clearly expressed how virtual care significantly reduced the time and planning required for appointments. One person compared it with having to make a 4-hour drive each way to the central clinic:

I don’t have to take a full day off of work and even though my husband attends all my appointments he doesn’t have to take full days off of work either and then we don’t have to try to, cause we’ve got animals, we don’t have to try to really worry about if they’re going to be in the car all day, if we bring them with us. And then if we’re staying overnight just not have to worry about trying to find care for them if we don’t bring them with us.

For this patient, as for most people, the impact goes beyond distance and travel time. There are ripple effects across family, work, and social networks when having to commit an entire day, or even an overnight stay, to attend a medical appointment. Climate and time of year were additional factors promoting the convenience of virtual visits compared with, “...a five hour drive through the [mountain] pass in the middle of winter often times.” Another efficiency remarkable to patients was the structure of the visit, where they met with different clinical team members in sequence during a single encounter:

I find it very efficient to be able to conference with somebody and have your concerns addressed without you having to physically be in their office. I find that the timing and the appointment time is much, much better on the video things because they make a point of being there and so do you.

Clinicians also recognized the importance of convenience for patients. One nurse observed, “I’m just guessing here, but I would say 95% appreciate the video conference and not having to travel. Like they choose it now.”

Another form of convenience for providers was related to specific role requirements. For example, a pharmacist found that phone call encounters during COVID-19 restrictions were helpful for the discussions she needed to have with patients. As patients were now calling from home, they could easily access all their medications:

...on the phone, when I’m phoning them at home I say can you please go get your medications. They might be sitting in their chair or on the phone with me and their medications are in their room or somewhere else and I make them go get them because I need to confirm exactly what they’re taking.

### Health Care Network

One immediately important network for patients, understandably, was their health care providers outside the renal team. Although our focus in the study was the kidney care team, participants frequently mentioned other clinicians in relation to their needs and preferences, often referring to primary care, usually their family physician. For some, there was a very positive link between the kidney care team and primary care. Even if they were unaware of the details, they felt reassured that information flowed easily between the 2 parts of the health care system.

Ya, a really important part of the team too. It’s not just the kidney specialist because I really need someone here so that anything that has to actually be physically looked at for it should be done here instead of having to travel there anyway, so all of it has been through virtual because I have my own doctor here and they keep in touch with the clinic and they know everything that’s going on and watch my blood tests every month and everything. They check up on all the stuff.

Health care networks looked different for patients with comorbid conditions and more complex needs. One patient described an effective communication network involving the kidney care team, 2 other specialist teams managing a comorbid condition, and the primary care physician:

...they work in conjunction with each other. Dr. C is the doctor in Vancouver and anything that he does or reports that he gets or tests that he does, Dr. S [another specialist] is made aware of them as well. So they’re hand in glove really. And then my GP here in [hometown] gets the same report and even from the [other specialists], all of that has been forwarded to all of them so they know what’s going on.

Another variation in health care networks was seen for peritoneal dialysis patients and providers, where material elements, such as dialysis equipment and the need for hands-on patient education and training, were central. Dialysis care required a network to facilitate the delivery or replacement of supplies, as well as training on when and how these actions were necessary. One patient mentioned that supplies were delivered during COVID-19 restrictions and left on the doorstep. A clinician described nurses in some local communities who “can change transfer sets for us.” One of the nurses spoke about a coordinating role involving the nephrologist, nurses, local pharmacies, and families to ensure the timely delivery of dialysis supplies. She would also check that local nurses had the necessary support to assist dialysis patients. For those involved with dialysis, the introduction of virtual care required additional logistics and resources due to the essential material elements in their networks.

### Peer Support

Not all the patients in the study mentioned peer support as part of their network, although one participant did bring it up:

I remember, well, this lady who became a friend of ours, she had found out about her kidneys going south and this mutual friend asked us, my wife and I to talk to her cause she was just going ballistic thinking life was over. So we went up and talked to her and now she’s had a transplant and things are going good for her too.

The same participant also mentioned the support he and his family received from the Kidney Foundation of Canada, which provided subsidized accommodation in Vancouver during an intensive treatment period that required him to stay in the city.

### Content of the Encounter: Trust and Virtual Relationships

People requiring specialty care for kidney conditions maintain ongoing contact with their kidney care team over years or even decades, following a chronic care model. Patients and providers have regular interactions, and when a virtual option was introduced, most patient participants welcomed the change, finding little difference compared with face-to-face encounters. One participant said, “It’s pretty much the same thing said and done as if you were actually sitting in a doctor’s office” and another, “I mean it’s good to meet them in person and talk about your problems...but no, the video conference filled that gap really easily.” While they acknowledged a difference, participants in our study did not place much value on it: “I guess it’s a little bit nice seeing a person’s face and everything but either way it doesn’t bother me too much.”

This contrasts with the trade-off of not having to travel, as mentioned earlier.

It’s a lot of inconvenience to have a 15-20 min conference with a doctor and then turn around and come home again. You’ve absorbed a whole day, where if I have video conference I can get done in the morning and my day is my own.

For some providers, by contrast, the loss of face-to-face encounters was seen as eliminating an essential aspect of care. One physician described the trade-off this way:

I think we’re pretty satisfied with how we’re able to provide these video conferencing visits, and I think seeing the patient satisfaction too is a good thing. But I think we all recognize that it doesn’t make up for, or it’s not as good as seeing someone face-to-face.

Virtual visits were intended to replace in-person visits, and one provider involved with the technical side of implementation stated, “we pretty much follow the same template as if we’re doing in-person care.” However, a provider in a clinical management role identified a hierarchy of preference: “...The face-to-face visits are much more important and sometimes the video conference, it’s almost like a touch-base in between.”

The idea that video or phone encounters are substandard compared with face-to-face visits might be reinforced or complicated by other changes that came with switching to video encounters. One practitioner noted a change in the duration of visits: “...[N]ormally I would see a client for at least 30 minutes, if not maybe 45 minutes...virtually it’s more like 15 minutes.”

Both patient and clinician participants pointed out a correlation between the stability of illness and the need for in-person encounters. In other words, if a patient’s condition became unstable with concerning symptoms or entered a new phase of treatment, such as dialysis, in-person communication became much more important. It should be noted that this flexibility was already built into the kidney program’s practices.

A dietitian commented that video images of faces omitted a lot of visual information she would typically observe if seeing a patient in person:

...for me assessing someone, like looking at their stature or looking at their legs or their knees or their hands or how their watch fits on their wrist, like I can’t see that on video conferencing.... It tells me about muscle wasting or that type of thing or just how they’re, ya, muscle wasting, how nutritioned, how well-nourished they are.

One nurse pointed out a less obvious distinction between video and “face-to-face” contact: “Video versus face to face doesn’t change bad news and responses...[but] I just think they’re going to hear it better [face to face].”

### Sound and Vision

The innovation of the initial virtual kidney care project allowed patients to connect via video from a room in a health care facility in their home community. However, when public health orders related to COVID-19 restricted hospital visitations, all virtual appointments were conducted by phone for the first time. In this context, both patient and provider participants expressed a preference for video over phone visits. One patient commented, “...You get all the information but it’s so much easier to actually see the person talking to you” and another said, “I think just as humans we like to see each other. So much of our communication is visual.” Providers noted that the visual element helped them gather more information about patients. One nurse who followed transplant patients explained, “...When I see their face I remember their case better than if I’m just talking to them on the phone.” A dialysis nurse added, “...I would say on the phone is even worse because you can’t judge their reaction.”

Virtual visits change many of the ways providers and patients are accustomed to interacting in an encounter, and the loss of sensory data during phone visits led to feelings of discomfort or awkwardness for team members, especially when trying to hit the right conversational tone or (re)establish a relational connection. One nurse noted the shift:

...It was so different when it was just over the phone. You don’t tend to ask those questions. It’s not as easy to just ask someone about, like you can’t just work it into the conversation in the same way...The relationship is different, I can’t explain it very well.

As we explored with participants the differences in experiencing virtual encounters, we found that patient participants did not report feeling disconnected or that the encounters were impersonal. Instead, patients consistently described how much they valued the team’s availability, their willingness to accommodate patient needs, and how approachable they were. Patients reported still feeling heard by the team and being seen as individuals. One said, “They give me their full attention every time we’re on the video screen and they seem genuinely interested in what’s going on with me. I haven’t seen it go the wrong way as far as the quality of care” and another, “They’re interested in what you’re doing, and they ask about more than just technical information.”

### Viewing the Team

The patient participants described a sense of trust in the clinical team, where multiple individuals showed interest in their health, symptoms, and personhood, both in person and virtually. The patient participants described comprehensive care experiences that extended beyond the virtual encounter and seemed to view the team as a cohesive unit. By contrast, providers described a more significant shift in their experience of the encounter and how it impacted their one-on-one interactions with patients when conducting visits virtually rather than in person. One physician put it that,

...it’s more focused on how is your blood pressure, are you taking your medication, do you have fatigue, shortness of breath, any problems with passing urine? I don’t say how are you doing, you look really good today, looks like you got your hair done, oh wow, okay, it’s your son getting married, that’s lovely.

In this study, however, patients described trusting the team and knowing they could rely more or less on the team’s resources depending on the trajectory and severity of their condition. One patient, for example, said “...a quarterly touch base or anybody who will respond to an email in between is fine with me. I’ve become less dependent on them; I don’t feel quite so terrified anymore. I’ve adapted to my life.” Another participant described how reassuring it was to know that the team would advocate for their care if needed.

My general practitioner is great, but I’d have to tell you that he’s far too busy. If I had to go to Emergency, I don’t fancy my chances. I sat in Emergency for 5 hours after being told by him that my potassium was super high and I sat there and waited for 5 hours because I looked healthy and I don’t fancy my chances. I feel that if I were to send a phone call or a message to them that they would phone ahead and pave my passage.

This team already had an existing interdisciplinary approach to supporting patients before implementing virtual encounters. For example, there is a “reach-in” mechanism where patients can phone a team member or email a provider with a question without needing to make an appointment. Patients expressed how much they valued knowing they could access the team when needed: “...if I ever have any questions that I need to ask them directly, I can phone the clinic and they will either find out themselves or talk to the doctor and get back to me within a day at the latest.” This enabled them to take initiative in their own care: “...it’s good to have the opportunity to...instead of having to make an appointment and waiting to speak to somebody, you can actually just call.”

A second critical factor in patients’ successful acceptance of virtual visits was the preexisting relationship they had with the kidney care team. Patients acknowledged that they felt comfortable during virtual visits because they already knew the team through face-to-face encounters.

Because I have had a relationship with them before this, everything moving virtual that it hasn’t had an impact on me. I certainly might be wary if I was new to this.

I know these people so talking to them on the phone is okay but if I was new I don’t know what my experience would’ve been.

Another participant reflected on the value of in-person encounters, saying, “It was a great opportunity to actually talk to them face to face so that now when I talk to them on the phone I kind of know who I’m talking to.”

However, a physician expressed that from the team’s perspective, new patients who had not experienced in-person visits might not feel the same sense of connection:

Now the patients that we’ve never seen before we all just blend in for them. Because it’s just three or four different people that step in and out of in front of their screen. I don’t want to say that maybe our message is not so believable anymore but we’re just a face now.

### Dialysis: In the Real World

Knowing patients is vital for providers, as it influences how they plan care, including follow-up or monitoring of patient conditions. It was notable that clinicians working with peritoneal dialysis patients were particularly concerned with enabling patients to use equipment to manage their dialysis at home. This raised broader implications about understanding each patient’s unique circumstances and needs:

It’s just me and them and you do get a good idea about how they learn, for one thing, and also you learn some things about their home situation and their life and their support system, that sort of thing. So those people who...I follow up and I’m talking to them frequently. So you have that established relationship. For those people then to do virtual assessment it’s easier because you have a good idea of what their baseline is, what they would normally look like, sound like, what their numbers normally would be. But for people who you don’t have that, for instance, a patient that I haven’t trained, that I’ve only seen virtually, then I’m lacking all that information and that makes it much more challenging.

A nurse working with dialysis patients also highlighted the importance of bodily presence and materials in providing care, especially when patients needed to learn hands-on skills for the correct use of equipment at home. One nurse described teaching patients about peritoneal dialysis using a dummy:

...so we can show them what it looks like, what it feels like, you know, where it sits in the belly. So I mean we can show them on video but they just don’t get the same hands-on and they just don’t get a feel for it so just not the same. It’s impersonal, right.

This nurse believed that the time spent teaching skills also provided valuable insights into understanding a patient’s circumstances:

So I guess we have the good idea because of the face to face contact we’ve had in the past so we start with [peritoneal dialysis] PD training and I’ve spent four days with the patient. Usually with their family member as well as teaching them. So it’s just me and them and you do get a good idea about how they learn, for one thing, and also you learn some things about their home situation and their life and their support system, that sort of thing.

Providers in this study recognized that achieving lasting satisfaction with virtual encounters may require learning new skills beyond those typically addressed in standard guidelines for virtual care. Provider job satisfaction is an important factor that contributes to the quality of care [18]. One physician noted, “All these little cues of reassurance and compassion, it’s harder. Because we see a face, we don’t see a person...I don’t have the same level of gratification with virtual appointments.” Providers identified the need to find ways to ensure their assessments were both complete and genuine. One nurse stated:

I like to see a face and I recognize faces, so I’m having a hard time if I haven’t seen a person for a year, oh man, what did they look like, you know. And then if they were to come in and say oh hi and I’m like who are you?...In virtual care you do see a face but it’s just not, it’s incomplete I guess is what I would say. You do not have the same assessment, an incomplete assessment.

A physician also expressed concern about the sense of incompleteness compared with in-person care, framing this as an ongoing challenge in adapting to virtual care:

...even though in various jurisdictions there are various levels of experience of virtual care, we’re still learning from it. It’s like an element of artificialness to it...So many other things that are going in people’s lives, they want to share that with us and that’s part of their connection with us and it can be better virtually, I’m not saying it’s not. It can be, we just haven’t developed that. We haven’t learned to make the virtual experience be closer to the human experience.

## Discussion

For the purposes of analysis, we divided our findings into 2 themes: structure and content. The introduction of virtual care, along with the shift from video appointments to phone consultations due to COVID-19, created ripple effects in the context of clinical encounters. While patients highlighted the convenience of virtual appointments, they also adapted their networks of care, managing hybrid combinations involving local primary care providers and informal support systems. Patients reported overall satisfaction with the content of virtual encounters and felt that team collaboration continued, even though providers perceived this collaboration as more fragmented. However, providers expressed concerns about constrained communication and the loss of the more holistic assessments typically made during in-person encounters. Another issue arose regarding privacy and confidentiality norms, which are usually controlled by professionals in a traditional clinic setting but were disrupted when patients could join appointments from virtually any location. In the discussion, we interpret various aspects of the encounter, keeping in mind that it is a multidimensional experience. We used broad themes of structure and content as a heuristic to give basic shape to our data. However, in practice, encounters are shaped by a dialectical interchange between both sets of factors. For example, convenience can be seen not only as a matter of time management but also as a sense of control over one’s time and resources. From the patients’ perspective, there was a high degree of satisfaction with virtual care, though some expressed a preference for in-person visits when managing increases in the complexity or severity of illness. When participants indicated preferences between modes of communication, the video was generally favored over the telephone. On the provider side, concerns were raised about the impact of virtual care on assessment and the relational tone of encounters with patients.

Virtual care is inherently hybrid, consisting of a mix of physical presence, unshared spaces, and communication technology that bridges the distance between 2 bodies in time. While this is often taken for granted, it highlights, from a phenomenological perspective, the various ways in which physical presence and separate spaces are interconnected by clinicians and patients. Paying close attention to the phenomenon allows for the recognition of subtle effects on behavior and power dynamics in clinical encounters—whether positive or negative, depending on the expectations and values of the participants. Recent phenomenological research on health and health care, such as Havi Carel’s [19] account of experiencing progressive respiratory disease, has shown the value of carefully examining the structures of experience.

Patient and provider perspectives revealed a marked contrast in the level of concern about the complications of remote communication. Patients felt that virtual visits met their needs, with few concerns raised. While the differences between in-person and virtual visits seemed intangible to patients, those in our study had been part of the kidney care program long enough to compare their experiences: attending the clinic in person, switching on the video in a local health care facility, or using the phone. Past experiences shape expectations in a health care encounter [20], and patients with an established relationship with a provider or team may have more success with virtual visits [13,21,22]. This may also reflect the foundational level of trust patients had in the kidney care team, which was further strengthened by the various “reach-in” features available. These allowed patients to call or email team members to have questions answered or seek advice outside of their scheduled appointments.

While patients still highly valued the ability to have in-person visits, whether scheduled in advance or made available if their condition changed, the need for an in-person encounter was weighed against the convenience of a virtual one. Other recent studies on the increase in virtual care usage have also highlighted the importance of balancing the complexity of illness or severity of symptoms with the patient’s desire to have in-person care remain an option [23-25].

Both patients and providers described a hierarchy of preferences for encounters, with face-to-face interactions (either in-person or via video) being preferred over telephone consultations. This preference has been noted in other studies as well [26], underscoring what may be lost without visual cues and sensory data. However, this preference for video consultations contrasts with findings in other research [11], where family practitioners favored shifting to telephone consultations. Participants’ language when describing their virtual care experiences reflects an awareness of the distinctions between in-person, video, and telephone encounters. “In-person” encounters clearly referred to times when both patient and provider were physically present in the same room. “Face-to-face” encounters, by contrast, were more ambiguous and could refer to either in-person or video appointments, but not telephone visits. Additionally, a distinction was made based on whether a visit required a “hands-on” component, highlighting moments when touch or manual dexterity was involved, such as when using a mannequin for learning peritoneal dialysis.

Provider participants in the study acknowledged the convenience virtual visits offered to patients but expressed greater ambivalence about virtual encounters. Zhang et al [27], in a literature review on the effects of virtual care on clinical reasoning, described an increase in ambiguity for clinicians. The ways in which clinicians synthesize a range of subjective and objective data were seen as becoming less integrated, with the style of interviewing often reverting to a more rudimentary and impersonal approach, resembling a checklist of questions about symptoms. One participant gave an example of observing a patient walking into the consulting room, immediately gaining an impression of their difficulty walking—an observation that he did not perceive as significant when it was only reported verbally by the patient.

The scope of attention narrows in on-screen encounters, and even more so in phone encounters. Clinician participants in our study described this as a reduction in the clinical horizon of attention. While pertinent information was not necessarily missed, the range of available information—drawn from sensory perception, interaction, and responses to questions—was notably diminished. This, in turn, related to a sense of trust in the relationship as being continuous and reliable, a quality conveyed more fully by in-person encounters or carried over into virtual encounters based on recollections of previous in-person meetings. Findings from this study support Prasad’s [4] argument that virtual care reduces the amount of information available to clinicians, making them more reliant on their own judgment to form a complete picture.

In virtual visits, providers described attempts to fill gaps in information or assessments that would have been easily addressed in in-person visits. Some providers noted that their interviewing style became more rudimentary, impersonal, and focused solely on medical symptoms. Other recent studies on the accelerated adoption of virtual care due to the COVID-19 pandemic have reported similar findings. In a survey of 696 patients and caregivers in Manitoba, Canada, Halas et al [24] noted that virtual encounters were experienced as more transactional. While patients did not report noticing or being concerned by these changes, our data revealed a tendency among patient participants to refer to their “numbers”—a shorthand for their kidney functioning—adopting a medical perspective that views measurable data as an objective indicator of their health status. This phenomenon is not solely a result of remote communication; it may also reflect how patients experience illness as both subjective and objective [28]. Patients might prioritize certain aspects of their health over others if clinicians, adjusting their focus based on a blend of subjective and objective data, have a narrowed horizon of attention in virtual compared with in-person encounters.

“The medium is the message,” as McLuhan [29] famously proposed, and virtual visits alter the meaning and significance of a health care encounter. For providers, the difference lay not only in the absence of the full physical presence of the patient but also in the varied settings where encounters now take place. Patients could participate in virtual care from home, their cars pulled over in a school parking lot, airport lounges, or even their workplaces. Virtual care transforms a medical office visit into a more routine, everyday encounter, removing the “ceremony” of the clinic [30]. Some of the unease expressed by provider participants in describing changes with virtual care may stem from the need to renegotiate their sense of legitimacy in a medium that does not readily convey authority or, necessarily, professionalism.

The shift in the locus of control during virtual encounters is a subtle aspect not yet fully explored in the literature. Virtual care toolkits emphasize provider attire, demeanor, privacy, and control over what is visible within the camera’s view. While this advice is typically placed under headings related to etiquette or privacy, it may also implicitly reinforce elements of power or legitimacy. Taken further, this may also influence how providers approach an encounter. Amid the many cognitive biases they try to identify in themselves, they may unconsciously reinforce the biomedical aspects of the encounter—not only to compensate for the objective data typically gathered during in-person visits but also to reassure the patient that this is a “real” medical interaction.

This study provides a nuanced understanding of virtual care encounters as experienced by kidney care providers and patients with chronic renal failure in a large, northern, sparsely populated region. One limitation is the specific setting, which may limit generalizability. Additionally, as with most qualitative research, the small sample size focuses on capturing detailed accounts of experiences rather than achieving statistical power. One implication of the small sample size is that we did not collect detailed information about participants’ indigeneity, socioeconomic status, or other characteristics, as these could potentially identify individuals in this rural setting. Instead of being directly transferable to similar populations, this study is intended to encourage those engaged in virtual care to consider new perspectives on the nature of the encounter.

By exploring both provider and patient experiences with virtual care, this study adds nuance and complexity to the often simplistic narratives of virtual visits’ convenience. It is important to remember that, at its core, virtual care is simply a form of communication between individuals, all of whom ultimately seek to have their bodily needs met and to find relief from suffering. Virtual care is inherently hybrid, and changes in how care is delivered create ripple effects across relationships and networks of care. Against this backdrop, it is well-established that patients and providers often have differing preferences regarding health care delivery [31]. As virtual care plays an increasingly significant role in improving access for patients facing geographical and financial barriers, further exploration of the unintended consequences of altering the medium of care delivery is crucial. Virtual care opens a long-closed gate, allowing patients to access care with minimal disruption to their everyday lives. However, it also raises new questions about legitimacy, power, and trust in health care encounters. This study contributes to the growing body of literature on virtual care post–COVID-19 by encouraging researchers and clinicians to consider the network of physical, social, and emotional aspects of people’s lives that are altered by a shift in the communication medium.

## Appendix

Multimedia Appendix 1 Interview guide used in the study for patient interviews.

Multimedia Appendix 2 Interview guide used with provider participants.

## Data Availability

The data sets generated during or analyzed during this study are available from the corresponding author upon reasonable request.
