# Supplementary material for: Effects of Virtual Care on Patient and Provider Experience of the Clinical Encounter: Qualitative Hermeneutic Study
Source: J Med Internet Res. 2024 Nov 26;26:e52552. doi: 10.2196/52552 (PMC11632281; doi:10.2196/52552)
Supplement: Multimedia Appendix 1 [file jmir_v26i1e52552_app1.docx]

**A Hermeneutic Perspective on Implementing Virtual Kidney Care in Northern BC**

**INTERVIEW QUESTIONS - PATIENTS**

*This research project has been created to study the processes through which the Virtual Kidney Care service has been and continues to be implemented. We understand that many processes have been disrupted by COVID-19, and we’re interested in the ways in which you experienced your kidney care before and how you are finding it now.*

1. Can you please tell me how you came to receive your care from the Kidney Care Service?

(Additional/Clarifying: What kind of conversations; joint decision-making? How long have you been part of the service, which team members do you talk with most, how are decisions made between the team and yourself regarding your care)

1. How was the idea of a virtual consultation first presented?
2. Would you tell me about a time (or situation) that would show what it was like for you to first receive care over video/telephone?

(Additional/Clarifying: What else was going on at the time?; How did you get through any technical issues? What do you find easier or different now compared to your first virtual visits?)

1. In what ways has that care changed over time, especially since March 2020?

(Additional/Clarifying: [re virtual care] What kind of conversations; joint decision-making? Who have you been talking to/receiving care from?/Give an example?- How did this change since mid-March 2020? )

1. Since March 2020, what has it been like to interact with the kidney team members? Can you tell me about a specific situation – to let me see what it has been like?

(Additional/Clarifying: Where has the care been received? Did you need to travel? Has the mode changed (e.g. video to telephone) and what is that like? Have you been more comfortable (less comfortable changing from the hospital/clinic to your home setting? What has made it more/less comfortable?

What do you hope will continue or not continue in terms of your visits or care?)

1. In what ways (if any) have your interactions with and care from your kidney care team changed since you’ve been receiving your care by distance? In what ways (if any) have your health changed?

(Additional/Clarifying: Developing a trusting relationship with your doctor/ nurses etc. is often thought to be important. Are you able to be open and as trusting over distance (phone/video). In what ways do you think it has influenced your care/how you are feeling about… Any changes in quality of care?)

1. What advice might you give to patients who were just about to start receiving kidney care in this region / by this team /in this way?
2. As a user of healthcare services, can you think of any comparable experiences with some change that you were asked to take on as part of your care or treatment? (Could be a change in technology, as here, or a change in treatment, or new information about your healthcare needs)

If there is one thing you would want us to know about what is important in receiving care for your kidney problems in this way, what would it be?

Note: Throughout the interview listen for and explore: prompting of changes, relationship, context, temporality, dialogue, expanding understanding, interpretation and connecting differing perspectives – and listen for what has been ‘shut down’ or ‘opened’ through the process of implementing.
