# Supplementary material for: Effects of Virtual Care on Patient and Provider Experience of the Clinical Encounter: Qualitative Hermeneutic Study
Source: J Med Internet Res. 2024 Nov 26;26:e52552. doi: 10.2196/52552 (PMC11632281; doi:10.2196/52552)
Supplement: Multimedia Appendix 2 [file jmir_v26i1e52552_app2.docx]

**A Hermeneutic Perspective on Implementing Virtual Kidney Care in Northern BC**

**DRAFT INTERVIEW QUESTIONS - PROVIDERS**

*This research project has been created to study the processes through which the Virtual Kidney Care service has been and continues to be implemented. We understand that many processes have been disrupted by COVID-19, and we’re interested in the ways in which you experienced providing the implementing of virtual kidney care and how you are finding implementing it now?.*

1. Can you please tell me about when you began work with patients virtually? What is your role?

(Additional: How has your role changed?)

1. How were you introduced to the implementation of virtual care before you presented it to patients?
2. Can you tell me about a time (or situation) that would show what it was like to plan for providing virtual kidney care?
3. How did you present virtual care to the patients? How was it received? How did you move through any areas of confusion or difficulty?
4. Would you tell me about a time (or situation) that would show what it was like for you to first provide care over video/telephone?

(Additional/Clarifying: What else was going on at the time?; How did you get through any technical issues? Is it easier now or not? What has helped or not?)

1. What has it been like for you to provide kidney care virtually? How has your practice changed with COVID-19?

(Additional/Clarifying: Has the mode changed (e.g. video to telephone) and what is that like? Have the patients you work with changed in any way because of virtual care? Give an example? What do you do now that is different (or the same) as before?)

1. Since COVID-19 what has it been like to interact with the patients? Can you tell me about a specific situation – to let me see what it has been like?

(Additional/Clarifying: What kind of changes in conversations; how might changes in joint decision-making been prompted by the virtual care? What do you hope will continue or not continue in terms of patient visits or care?)

1. In what ways has your interactions with and care from your kidney care team changed (and kept changing) since you’ve been providing kidney services by distance – and since COVID-19? What effect do you think it has had (is having) on patients ?

(Additional/Clarifying: Developing a trusting relationship with patients etc. is often thought to be important. In what ways do you think it has influenced your care/how you are feeling about…. Picking up on the little things? How are you able to do that – and how has your care changed?)

1. We are interested in what we might learn about the process and experience of implementation from this particular project. Can you think of any other examples of implementing new processes, new technology, new knowledge that you have been involved with? If so, how did they compare to your experience with the Virtual Kidney care service?
2. What advice might you give to other [type of provider] who were just about to start providing kidney care in this way?
3. If there is one thing you would want us to know about what is important in implementing virtual kidney care or a service like it what would it be?

(Implementing of the service vs their own practices in implementing teleconsults - (what influences changing the system vs what influences changing their own practice).

Note: Throughout the interview listen for and explore: implementing - relationship, context, temporality, dialogue, expanding understanding, interpretation and connecting differing perspectives – and listen for what has been ‘shut down’ or ‘opened’ through the process of implementing the virtual kidney care services and its changes.
